# Effects of cognitive bias modification on social anxiety: A meta-analysis

**DOI:** 10.1371/journal.pone.0175107

**Published:** 2017-04-06

**Authors:** Haining Liu, Xianwen Li, Buxin Han, Xiaoqian Liu

**Affiliations:** 1 Key Lab of Mental Health, Institute of Psychology, Chinese Academy of Sciences, Beijing, China; 2 Psychology Department of Chengde Medical University, Chengde, China; 3 University of Chinese Academy of Sciences, Beijing, China; 4 School of Nursing, Nanjing Medical University, Nanjing, China; University of Oxford, UNITED KINGDOM

## Abstract

**Background:**

Cognitive bias modification (CBM), a set of techniques for modifying bias in information processing—is considered a novel intervention for social anxiety disorder (SAD), which has drawn considerable interest from researchers. However, the effects of CBM on SAD are not consistent. Some studies have demonstrated significant positive effects compared to control groups, while others have found no such effects.

**Aims:**

We conducted a meta-analysis aimed at quantitatively assessing the effects of CBM on SAD at post-test.

**Method:**

Through a systematic literature search by two independent raters, 34 articles (36 randomized studies) including 2,550 participants were identified. A multilevel modeling approach was employed to assess the effects of CBM on SAD, and to explore the potentially crucial procedures and sample characteristics that enhance the effectiveness of benign training.

**Results:**

In general, there were small but significant effects of CBM on the primary symptoms of SAD (*g* = 0.17), cognitive bias (CB) toward threat (*g* = 0.32), and reactivity in stressful situations (*g* = 0.25), but non-significant effects on secondary symptoms. However, the interpretation modification program was more effective than was attentional bias modification in reducing SAD primary symptoms and negative CB. Laboratory training procedures produced larger primary symptom reductions compared to Internet-based training, whereas the percentage of contingency and feedback about training performance boosted cognitive effects only. Finally, the following groups were more likely to benefit from CBM: younger participants (primary symptoms and cognitive effects), women (primary symptom effects), and samples with stronger CB (stressor effects). The quality of the randomized controlled trials was less than desirable, as there was some indication of publication bias in our study.

**Conclusions:**

Current findings broadly supported cognitive theories of SAD that consider a bidirectional or mutually reinforcing relationship between symptoms and CBs. However, the small therapeutic effect observed here indicates that it is necessary to develop more reliable and efficient CBM interventions that are specific to SAD.

## Introduction

Social anxiety disorder (SAD), also referred to as social phobia [1], is a condition marked by sustained fear and avoidance of certain or most social situations because of concerns about evaluation by others. Large-scale epidemiological studies have shown that SAD is one of the most prevalent psychological disorders. The findings of the World Health Organization (WHO) World Mental Health (WMH) Survey Initiative showed that lifelong social phobias are generally prevalent in both developed (15.9%) and developing (14.3%) countries [2]. A greater lifetime risk of SAD was found in people in developing countries as compared to those in developed countries. Additionally, SAD is common among young adults, and it has an early onset age—about 50% of the cases show symptoms by the age of 11 and about 80% of the cases by the age of 20 [3]. Women are disproportionately likely to develop SAD relative to men according to a National Epidemiology Survey on Alcohol and Related Conditions (NESARC) study [4–6]. Moreover, SAD is usually comorbid with other mental health problems, such as depression, specific phobias, or another anxiety disorder, which are often associated with serious impairment in educational, occupational, and social functioning [7]. Therefore, it is of great importance to develop more effective, accessible, and acceptable treatments for individuals with SAD.

There are diverse explanations for the etiology of SAD from a psychological perspective. For example, psychodynamic conceptualizations of social anxiety encompass numerous schools of thought, including instinctual models, ego-psychology, defense mechanisms, object relationships, and attachment theories [8, 9]. Symptoms of SAD are often believed to originate from childhood traumatic experiences that made the patient feel miserable or abandoned by their parents [10]. Apart from these psychodynamic approaches, researchers adopting an interpersonal approach have assumed that social skill deficits are one of the principal factors underlying SAD [11]. If individuals with SAD feel threatened in a situation, this situation may elicit self-defensive behaviors to guard against negative evaluations. Similarly, the cumulative interpersonal risk model of social anxiety in youth maintains that social skills impairment is one of many interpersonal risk factors for SAD [12]. However, in recent years, emerging cognitive theories—such as the information processing perspective—have maintained that social anxiety is associated with cognitive biases (CBs) in the processing of emotionally congruent information, which in turn play a key role in the onset and development of SAD [13–17]. Cognitive models of SAD propose that individuals with higher levels of social anxiety automatically and selectively attend to socially threatening information (attention bias) and interpret emotionally ambiguous events as threatening (interpretation bias) [18,19]. For instance, in novel social scenarios, individuals with SAD are inclined to perceive social threat cues (i.e., someone frowning) and to interpret it as a signal that they are behaving poorly.

Cognitive bias modification (CBM) is a novel experimental technique, built on these cognitive theories of SAD, aimed at reducing negative CBs and thereby diminishing anxiety susceptibility and symptoms. A typical CBM paradigm is the modified dot probe task [20], which is based on MacLeod’s (1986) classic experiment. In a traditional dot-probe task, a pair of stimuli (i.e., a threatening face or word and a neutral counterpart) is presented on a computer screen simultaneously. Immediately following these cueing stimuli, a target stimulus (i.e., one or two dots) appears in the location previously occupied by one of the cueing stimuli. The participants are required to judge the location of the target as quickly and as accurately as possible. Responding faster to the target that replaced the threatening stimulus than to the target that replaced the neutral stimulus indicates attentional vigilance toward threatening information. The Attentional Bias Modification (ABM), paradigm subsequently modified the dot-probe task to direct participants’ attention away from threat, with the probe almost always replacing a benign stimulus (i.e., 80%–100% of the trials).

The interpretation bias paradigm is another typical CBM paradigm; while similar to ABM, it uses more complicated stimulus materials, such as the application of ambiguous sentences or paragraphs. For example, a series of ambiguous sentences are presented to participants, and they are requested to disambiguate the emotional valence of the stimuli towards a positive (or neutral) or negative interpretation. For instance, participants are presented with a word conveying either a threat interpretation (i.e., “embarrassing”) or a positive interpretation (i.e., “amusing”) before the following ambiguous sentence: “After you said something, people laughed.” They are then asked to indicate whether the word and sentence were relevant or irrelevant [21]. The Interpretation Modification Program (CBM-I) encourages participants to develop a benign interpretation of the ambiguous experimental materials. In other words, when participants generate a benign endorsement or refuse threat endorsement of ambiguous material, they are given positive feedback (i.e., ‘‘Correct!”), otherwise negative feedback is presented (i.e., ‘‘Incorrect!”). There are a number of other CBM interventions, such as the approach-avoidance task (AAT) wherein participants push (approach) or pull (avoid) pictorial cues presented on a computer screen using a joystick. This task aims to train participants to approach positive pictures and avoid the negatives, in order to led to more positive mood and lower anxiety [22].

These therapies involve similar implicit cognitive retraining strategies to alter CB. There is growing evidence that CBM might lead to decreases in both self-reported and behavior-based measures of anxiety in socially anxious participants, including both clinically diagnosed [23–26] and subclinical samples [21,27,28]. However, over the past few years, other studies have offered some mixed results [29–32]. Specifically, findings have shown that compared with controls, the CBM group had no significant difference at post-test in mitigating CB or SAD symptoms [27, 30].

To date, only one previous meta-analysis has been conducted on CBM techniques; the study summarized the findings of 15 randomized studies on the use of ABM to reduce negative attention bias and social anxiety [33]. Their results showed a small reduction in SAD cardinal symptoms (*g* = 0.27), responses to speech challenges (*g* = 0.46), and attention bias (AB) (*g* = 0.30). To explore potential moderators, the researchers found that laboratory-delivered CBM yielded larger effect sizes (ESs) in SAD symptom reduction than it did in Internet-delivered treatments. Additionally, the experimental design and trait anxiety at pre-test moderated these effects. Although this quantitative review took an important step toward the assessment of cognitive theories of social anxiety, its narrow focus on ABM meant that many CBM (i.e., CBM-I) studies were excluded. A more comprehensive quantitative review that covers interpretation, attention biases, and social anxiety is needed to clarify unresolved questions about CBM and its underlying theory.

The present meta-analysis aimed to investigate the clinical effects of CBM on social anxiety systematically, in terms of both treatment effects (i.e., primary/secondary symptoms of SAD andreactivity in stressful situations) and crucial moderators that predict the degree of change. The first goal was to evaluate the degree to which certain variants of CBM can successfully relieve SAD symptoms and CBs. The assumption of the present study was that CBM can modify cognitive biases that contribute to SAD symptoms; indeed, a recent review has concluded that ABM can modify AB toward threat and reduce SAD symptoms [33]. However, it is still unclear to what extent other types of CBs can be modified to produce changes in SAD symptoms. The second goal was to identify the potential moderators of the effectiveness of CBM on SAD. Previous reviews and theories have suggested a number of potential factors affecting the efficacy of CBM, such as the clinical status of the study sample, CB, specific procedural details of the interventions (i.e., training contingency, or whether the target almost always replaced the neutral/positive probe or whether the replacement was random; feedback; training setting; and number of training session), the severity of SAD symptoms at baseline (i.e., baseline score on the Liebowitz Social Anxiety Scale), baseline CB, participants’ characteristics (i.e., age and gender ratio), and impact factor of publications [34–37]. The third and final goal was to evaluate the quality of included studies and identify potential publication bias. Specifically, the present study examined whether there is robust empirical evidence with strong methodological quality to support the clinical efficacy of CBM reported in these studies.

## Method

### Identification and selection of studies

With strict adherence to the PRISMA guidelines [38], the first and second authors searched the literature independently (see S1 Table). Records were identified by searching multiple literature databases (see S1 Text), including ISI Web of Science, PubMed, PsycInfo, EMBASE and the Cochrane Collaboration's register of controlled trials, through December 2015. The following combinations of keywords were used in this search: “cognitive bias modification,” “attention* bias modification,” “interpret* bias modification,” “attention* training,” “bias experimental manipulation,” and “bias training” paired with “social phobi*”or “social anxi*” (see S1 Text). There were no language restrictions. The references within the most recent reviews on CBM for social anxiety disorders were also systematically searched [33, 39]. Unpublished studies were not included.

**Fig 1:**
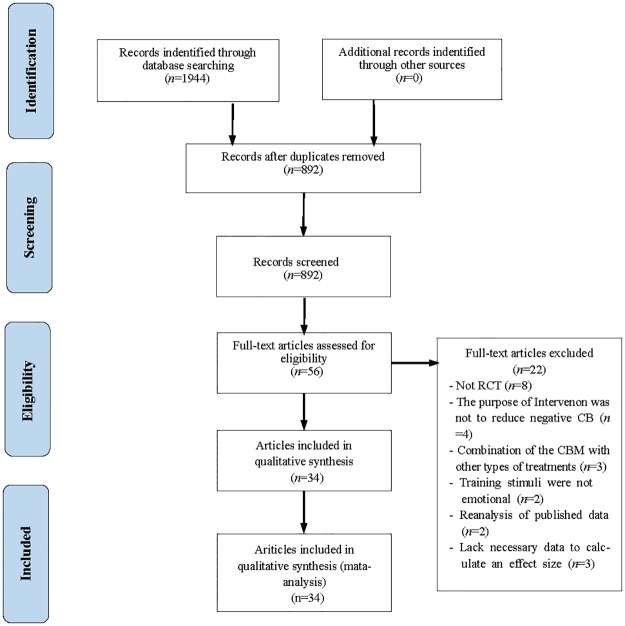
The PRISMA flow diagram of study selection [38].

A total of 1,944 records were identified (see Fig 1 for details). After removal of duplicated records, the titles and abstracts of the remaining articles were screened. After eliminating obviously irrelevant publications, 56 potentially relevant articles were retained and their full texts were reviewed for incorporation into the meta-analysis. Studies were included in the review if: (a) a randomized controlled trial (RCT) was used; (b) the therapeutic effect of CBM was isolated and not merged with other treatments; (c) the study was designed specifically to manipulate CB for the purpose of alleviating SAD symptoms and emotional susceptibility among patients with SAD or in subclinical populations; (d) at least one control group was included (i.e., a placebo control); (e) social-anxiety-relevant symptoms (i.e., SAD symptoms, such as self-reported reactivity in stressful situations or clinician-administered symptom assessment) were assessed at least once after training; (f) positive or negative emotionally relevant stimuli were used as training stimuli (i.e., strongly valenced words, facial expressions); and (g) sufficient data were available to compute ESs.

Some studies included two or even more control groups (i.e., attend threat, the sham intervention, or waiting-list groups) [26–28, 32]. We selected the group that was closest to the placebo condition (i.e., sham intervention group) to calculate ESs, as this was the most common approach used for control groups in CBM studies. Two studies used a four-groups design, including two experimental and two control groups, to examine the effectiveness of CBM [40, 41]. In addition, one study reported a six-group design—three experimental and three control groups [42]. For such studies, only the data sets that were related to the standard CBM and control conditions were extracted. Finally, with regard to findings for extended samples [43, 44] based on original studies [23, 45], we only chose the datasets reported in the original studies [23,45], as these offered more information about possible moderators.

### Quality assessment and data abstraction

We used the risk of bias assessment tool formulated by the Cochrane Collaboration [46] to evaluate the methodological quality of the studies included in the meta-analysis. Five criteria from this tool were chosen to examine sources of bias in RCTs: (1) generation of adequate random allocation sequence, (2) allocation concealment prior to assignment, (3) blinding of participants to information about the allocated intervention, (4) blinding of outcome assessors to information about the allocated intervention, and (5) dealing with incomplete outcome data. The last criterion was met if there were no missing data or the missing data were managed using an intent-to-treat approach. The quality assessment of the studies was implemented by two independent authors (Liu and Li). Conflicting opinions were resolved via discussion; if this did not resolve the conflict, the authors sought the advice of the senior author (Han).

Information about following characteristics of the included studies was collected (see Table 1):

**Table 1:** Selective characteristics of included studies.

Study	Clinical status of participant	N	Mean age	%—total Female	N-total sessions	Type of training materials	Training setting	Bias	Feedback (y/n)	Stressor (y/n)	Measures
Amir, et al. (2008) [54]	Subclinical	94	19	51	1	Disgust faces / neutral	Lab	A	n	y	STAI–S, Spatial cueing task
Amir, et al. (2009) [23]	Clinical diagnosis	44	29	59	8	Disgust faces / neutral	Lab	A	n	n	BDI–II, HAM–D, LSAS, SPAI, STAI–T, Dot-probe task
Amir, et al. (2010) [55]	Subclinical	57	19	53	1	Benign interpretation / threat	Lab	I	y	n	STAI, Spatial cueing task
Amir & Taylor (2012) [24]	Clinical diagnosis	49	31	71	12	Benign interpretation / threat	Lab	I	y	n	BDI-II, LSAS, SPAI, STAI-T, WSAP
Asnaani et al. (2014) [29]	Clinical diagnosis	43	28	75	2	Smiling faces / checkerboards	Lab	AA	n	y	BDI-II, IPSM, LSAS, SPIN, STAI-S
Beard & Amir, (2008) [21]	Subclinical	27	20	93	8	Disgust faces / neutral	Lab	I	n	n	BDI-II,SPAI-SP, STAI-T, WSAP
Beard, et al. (2011) [25]	Clinical diagnosis	32	37	75	8	Disgust faces / neutral + benign interpretation / threat	Lab	A+I	y	y	LSAS
Boettcher, et al. (2012) [56]	Clinical diagnosis	68	38	37	8	Disgust faces/neutral	Internet	A	y	n	BDI, LSAS, SIAS, SPS, Spatial cueing task
Boettcher, et al. (2013) [42]	Clinical diagnosis	129	38	64	14	Positive words / threat /neutral + happy faces / disgust / neutral	Internet	A	n	n	BAI, LSAS, MADRS, QOLI, SPS, Dot-probe task
Bunnell, et al. (2013) [31]	Clinical diagnosis	31	24	45	8	Disgust faces / neutral	Lab	A	n	y	BARS, BDI, BSPS, CGI, IST, LSAS, SPAI, UCT
Carlbring, et al. (2012) [45]	Clinical diagnosis	79	37	68	8	Disgust faces / neutral	Internet	A	n	n	BAI, LSAS, SIAS, SPS, SPSQ
Carleton, et al. (2015) [57]	Clinical diagnosis	108	36	58	8	Threat words / neutral	Lab/Internet	A	n	n	CES-D, SIPS, STAI-T
De Voogd, et al. (2014) [58]	Subclinical	32	15	50	2	Positive faces / negative	Lab	A	y	n	RCADS
Enock, et al., (2014) [27]	Subclinical	429	35	48	83	Threat faces /neutral	Smartphone	A	n	n	DASS, LSAS, PSWQ, SIAS, Dot-probe task
Heeren, et al. (2011) [59]	Clinical diagnosis	41	21	71	1	Disgust faces/neutral	Lab	A	n	y	BASA, VAS, Spatial cueing task
Heeren, et al. (2012) [26]	Clinical diagnosis	38	22	55	16	Positive faces / threat	Lab	A	n	y	BASA, FNE, LSAS, SCR, SUDS, Dot-probe task
Heeren, et al. (2015) [60]	Clinical diagnosis	61	26	81	2	Disgust faces /neutral	Lab	A	n	y	BASA, SUDS, Spatial cueing task
Hoppitt, et al. (2014) [61]	Subclinical	69	21	79	5	Benign interpretation / neutral	Internet	I	y	n	FNE, PANAS, STAI-S, STAI-T, Dot-probe task, ambiguous social scenarios
Julian, et al. (2012) [41]	Subclinical	56	23	68	1	Disgust faces / neutral	Lab	A	n	y	STAI-S, Spatial cueing task
Khalili-Torghabeh, et al. (2014) [62]	Subclinical	35	20	39	4	Benign interpretation / neutral	Lab	I	n	n	FNE, SADS, ambiguous social scenarios
Klumpp & Amir, (2010) [28]	Subclinical	87	18–22	72	1	Disgust faces / neutral	Lab	A	n	y	STAI-S
Li, et al. (2008) [63]	Subclinical	24	23	40	7	Positive faces / negative	Lab	A	n	n	FNE, SIAS, SPS, Dot-probe task
Maoz, et al. (2013) [64]	Subclinical	51	38	74	4	Angry faces / neutral	Lab	A	n	y	LSAS, STAI-S, Dot-probe task
McNally, et al. (2013) [32]	Subclinical	57	21	66	4	Disgust faces / happy	Lab	A	n	y	DASS, LSAS, PRCS, SPRS, Dot-probe task
Murphy, et al. (2007) [65]	Subclinical	44	40	75	1	Benign interpretation / neutral	Lab	I	y	y	STAI-S,ambiguous social scenarios
Neubauer, et al. (2013) [66]	Subclinical	57	26	53	8	Disgust faces / neutral	Internet	A	n	n	BDI, LSAS, SIAS, SPS, Dot-probe task
Nowalcowski, et al. (2015) [67]	Subclinical	48	32	44	1	Benign interpretation / neutral	Lab	I	y	y	PSP, SPIN, ambiguous social scenarios
Rapee, et al. (2013) [68]	Clinical diagnosis	134	23	77	12	Social words / neutral	Internet	A	n	y	DASS, SIAS, SPS, STAI-S
Schmidt, et al. (2009) [69]	Clinical diagnosis	36	14	70	8	Disgust faces / neutral	Internet	A	n	n	BDI, BSPS, LSAS, SPAI, STAI–T
Sportel, et al. (2013) [70]	Clinical diagnosis	156	11	53	20	Positive faces or words / neutral / threat benign + interpretation	Internet	A+I	n	n	RCADS
Taylor & Amir (2012) [71]	Subclinical	44	20	72	1	Positive faces / neutral	Lab	AA	n	y	STAI-S
Vassilopoulos, et al. (2009) [72]	Subclinical	43	11	47	3	Benign interpretation / negative	Lab	I	y	n	CDI, SASC, ambiguous social scenarios
Vassilopoulos, et al. (2013) [73]	Unselected	153	11	50	4	Benign interpretation / negative	Lab	I	y	n	CDI, SASC, ambiguous social scenarios
Vassilopoulos & Brouzos (2015) [74]	Subclinical	94	11	60	1	Benign interpretation / negative	Lab	I	y	y	CDI, SASC, ambiguous social scenarios

Notes. DSM-IV = Diagnostic and Statistical Manual of Mental Disorders; SAD = Social Anxiety Disorder; SP = Social Phobia; A = attentional bias; BDI-II = Beck Depression Inventory-II; HAM-D = Hamilton Rating Scale for Depression; LSAS = Liebowitz Social Anxiety Scale; SPAI = Social Phobia and Anxiety Inventory; STAI-S/STAI-T = State-Trait Anxiety Inventory-State/State-Trait Anxiety Inventory-Trait; I = interpretation bias; WSAP = word–sentence association paradigm; SPAI-SP = Social Phobia and Anxiety Inventory—Social Phobia subscale; SIAS = Social Interaction Anxiety Scale; SPS = Social Phobia Scale; BAI = Beck Anxiety Inventory; MADRS = Montgomery-Asberg Depression Rating Scale; QOLI = Quality of Life Inventory; BARS = Behavioral Avoidance Rating Scale; BSPS = Brief Social Phobia Scale; CGI = Clinical Global Impression of Improvement; IST = Impromptu Speech Task; UCT = Unstructured Conversation Task; SPSQ = Social Phobia Screening Questionnaire; CES-D = Centre for Epidemiological Studies Depression Scale; SIPS = Social Interaction Phobia Scale; RCADS = The Revised Child Anxiety and Depression Scale; DASS = Depression, Anxiety, and Stress Scale; PSQW = Penn State Worry Questionnaire; BASA = Behavioral Assessment of Speech Anxiety; VAS = Visual Subclinical scales; SUDS = Subjective Units of Discomfort Scale; FNE = Fear of Negative Evaluation; SCR = skin conductance reactivity; PANAS = Positive and Negative Affect Schedule; SADS = Social Avoidance and Distress Scale; PRCS = Personal Report of Confidence as a Speaker; SPRS = Social Performance Rating Scale; PSP = Perception of Speech Performance; SPIN = Social Phobia Inventory; CDI = Children’s depression inventory; SASC = Social anxiety scale for children; AA = AAT Approach Bias; IPSM = Interpersonal Sensitivity Measurement

- *Publication information*: including identification information (author and year of publication), and the journal impact factor (through Web of Science) at the time of publication;
- *Characteristics of the study sample*: including source (clinical/subclinical), number of participants across different conditions, percentage of women per study, and seriousness of primary and secondary symptoms of social anxiety (i.e., the Liebowitz Social Anxiety Scale or Beck Depression Inventory score at baseline);
- *Characteristics of the CBM procedure*: including treatment dose (total number of sessions), type of training materials (i.e., type of threaten and non-threaten cues), training setting (laboratory, internet, or smartphone), type of bias intervention (i.e., ABM or CBM-I), the presence of a stressful task during the training program (yes or no), feedback on accuracy when the tasks were solved (yes or no), and the percentage of trials in the CBM condition in which cues were valid.

The outcome measures were classified into four categories (see S2 Table): (1) self-reported primary social anxiety symptoms at post-test; (2) negative CB at post-test; (3) reactivity in stressful situations (i.e., speech challenge); (4) secondary social anxiety symptoms, both self-reported and clinician-rated, at post-test (i.e., depression and general distress).

### Data analysis

For each between-group comparison, the ES (Cohen’s *d*) was calculated to indicate the differences between the CBM intervention and a control group at post-training. The ESs were calculated by subtracting the mean value of the CBM group from the mean value of the comparison group (at post-training), and dividing by the pooled standard deviations of both groups. Cohen’s *d* values of 0.2, 0.5, and 0.8 are considered small, medium, and large ESs, respectively [47]. Because some studies had small sample sizes, the corrected ES coefficient—Hedges’ g—was applied [48]. This can be interpreted like Cohen’s *d* [35].

We calculated the pooled mean ESs using Comprehensive Meta-Analysis (CMA; version 2.2.064 for Windows). As there were a number of measurements used to assess the symptoms, we classified the outcome measures as follows: SAD primary symptoms, CB, reactivity in stressful situations, and secondary symptoms (see S2 Table). When a study included several instruments measuring the same outcome, an average ES was calculated. When calculating averages, we weighted ESs by their sample size. If the studies did not report the means and standard deviations, we used the other statistics recommended by CMA 2.0, such as *t* values, sample sizes, and precise *p* values from independent groups. Additionally, we emailed the authors to inquire prudently whether there were any related unpublished data.

We also aimed to investigate how the baseline (i.e., before training) CB influenced the therapeutic benefits yielded by CBM to SAD symptoms, CB, and reactivity in stressful situations. Thus, we imputed a third data set of ESs and coded them as positive values to reflect a larger baseline CB in the CBM group relative to the control group. Only ESs for post-test and post-challenge were reported. Follow-up data were not considered because the duration differed considerably and participants in some studies underwent treatment while others did not. ESs were computed with random-effects models, which assume the observed estimates of treatment effects vary within-study (as with fixed-effects models) as well as between-studies, because this method is generally considered more conservative [49].

Homogeneity of ESs was tested using the *Q*-statistic and the *I*^*2*^-statistic [50]. A significant *Q*-statistic suggests that there is true heterogeneity in ESs exceeding the random error. The *I*^*2*^ quantifies the degree of heterogeneity in percentages, with higher values indicating higher heterogeneity. A value of 0% is regarded as no heterogeneity, while those of 25%, 50%, and 75% indicate low, moderate, and high heterogeneity, respectively [51]. When investigating categorical moderators, not only the heterogeneity within each group (*Q*_*w*_) but also the heterogeneity between groups (*Q*_*b*_) were evaluated. Significant between-group heterogeneity indicated that the moderators were significant.

Publication bias was examined using two methods: visually observing the funnel plots for the main outcome classification, and employing the trim and fill procedure of Duval–Tweedie to obtain a corrected ES [52]. Outliers were defined as an ES of ≥2.5 standard deviations (*SD*) above or below the average ES estimate in each dataset [53]. The robustness of the findings was assessed using sensitivity analyses, which involved comparing results when including and excluding outliers.

Categorical moderators were tested with subgroup analyses using a mixed-effects model. In this model, we investigated pooled studies within subgroups using a random-effects model, whereas significant differences between subgroups were examined using a fixed-effects model. If there were less than three studies within a subgroup, it was not reported. For continuous moderators, unrestricted maximum likelihood meta-regression analyses were conducted.

## Results

Thirty-four articles met the selection criteria, which involved a total of 36 RCTs with 2,550 participants. The number of training sessions varied from 1 to 83, with 7 RCTs including just one session. Seventeen RCTs used SAD samples that were diagnosed based on DSM-IV criteria, 18 subclinical samples, and 1 used unselected participants. In total, 25 interventions were carried out in a laboratory setting, 10 via the Internet, and 1 via smartphone. Twenty-two studies examined ABM, 10 examined CBM-I, 2 examined a combination of CBM methods, and 2 examined AAT.

### Methodological quality of included studies

**Fig 2:**
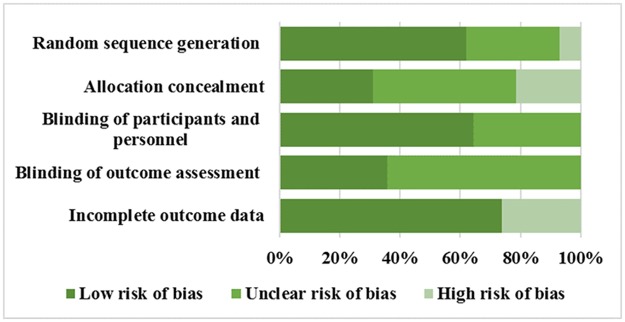
Risk of bias chart: Each item summarized according to the authors’ judgments of risk of bias presented as a percentage of all the included studies.

Overall, the quality of the included RCTs was heterogeneous. Half of the studies (18/36) met over three of the five quality criteria considered, while 5.6% (2/36) did not satisfy any quality criteria, and only 13.9% (5/36) satisfied all five criteria. Except for handling incomplete outcome data, a relatively large proportion of RCTs (41.7% for generating random sequence, 30.6% for concealing allocation, 19.4% for blinding of participants, and 33.3% for masking of assessors) did not report sufficient information for judging whether high or low risk of biases existed (please see Fig 2 for details).

### Meta-analysis of CBM effect on all outcome categories

#### SAD primary symptoms

**Main effect sizes**. CBM had small but significant effects on measures of SAD symptoms at post-test (*g* = 0.26, 95% CI: 0.09 to 0.43, *N* = 25, *Z* = 2.95, *P* = 0.003). There was strong evidence of heterogeneity across the included RCTs [*Q*(24) = 63.94, *P* < 0.001, *I*^*2*^ = 62.46%]. One study [69] was considered an outlier (using a cut-off of 2.5 *SD* from the average ES) [53]. With its removal, the effect was still significant but decreased (*g* = 0.17, 95% CI: 0.04 to 0.30, *N* = 24, *Z* = 2.52, *P* = 0.012) and the heterogeneity became non-significant [*Q*(23) = 33.92, *P* = 0.066, *I*^*2*^ = 32.20]. We removed this outlier study from further analyses. Furthermore, we conducted a sensitivity analysis wherein we removed each study at a time and computed the ESs for the remaining studies; overall, the results were not influenced by any one study.

**Subgroup and meta-regression analysis**. The subgroup analysis was used to test the influence of the categorical moderators. As shown in Table 2, for type of bias, effect sizes for CBM-I were significantly higher than they were for ABM on SAD symptoms. Additionally, studies based on a laboratory environment had significantly larger ESs than did those delivered via the Internet. Heterogeneity remained in the subclinical samples, showing that other possible major sources of heterogeneity might exist. The studies wherein CB was successfully modified had significantly larger ESs than did those wherein CB was not modified.

**Table 2:** Subgroup analysis for the primary symptoms of social anxiety disorder at post-test.

Moderator	*N*	*g*	95% CI	*Z*	*P*	*Q*_*w*_	*P*	*Q*_*b*_	*P*
Clinical status									
Diagnosed	13	0.11	-0.07 ~ 0.27	1.39	0.164	13.96	0.303	1.49	0.222
Subclinical	10	0.30	0.05 ~ 0.54	2.36	0.018	17.89	0.037		
**Bias**									
**Attention**	**14**	**0.01**	**-0.01 ~ 0.13**	**0.09**	**0.932**	**5.21**	**0.970**	**5.76**	**0.016**
**Interpretation**	**8**	**0.28**	**0.10 ~ 0.47**	**3.04**	**0.002**	**16.46**	**0.021**		
Stressor									
Y	9	0.16	-0.04 ~ 0.35	1.57	0.117	7.98	0.436	0.16	0.692
N	14	0.21	0.02 ~ 0.40	2.17	0.030	24.56	0.026		
N of training session									
One	3	0.37	0.004 ~ 0.74	1.98	0.05	2.29	0.318	1.08	0.298
More than one	20	0.16	0.02 ~ 0.31	2.20	0.028	28.26	0.079		
**Training setting**									
**Lab**	**15**	**0.38**	**0.20 ~ 0.57**	**4.03**	**0.000**	**17.62**	**0.225**	**10.21**	**0.001**
**Internet**	**7**	**-0.03**	**-0.21 ~ 0.14**	**-0.38**	**0.704**	**1.40**	**0.966**		
Feedback									
Y	7	0.33	0.06 ~ 0.60	2.38	0.017	9.61	0.142	1.71	0.191
N	16	0.12	-0.03 ~ 0.27	1.59	0.112	19.91	0.175		
**CB modified**									
**Y**	**12**	**0.30**	**0.09 to 0.51**	**2.82**	**0.005**	**22.00**	**0.024**	**4.99**	**0.025**
**N**	**10**	**0.001**	**-0.16 to 0.16**	**0.02**	**0.988**	**6.74**	**0.664**		

Meta-regressions were applied to examine the effect of continuous moderators. The results showed that the post-test ESs were significantly moderated by three variables (see Table 3). First, the percentage of women in the sample moderated ES; larger ESs were found in the sample that comprised a greater percentage of women. Second, participants’ age within studies showed a significant moderating effect, such that younger participants benefited the most from CBM. Finally, a significant, negative relationship was found between publication year and ES.

**Table 3:** Meta-regression for the outcome categories at post-test.

Outcomes	Moderators	*N*	*β*	*SE*	*Z*	*P*
SAD symptoms	Number of training trials per session	23	0.00004	0.0004	0.09	0.926
	*%* Contingency	23	0.01	0.006	1.76	0.079
	CB at baseline	18	0.69	0.46	1.51	0.132
	LSAS at baseline	12	- 0.001	0.01	-0.14	0.892
	BDI at baseline	9	0.02	0.02	0.70	0.486
	STAI-Trait at baseline	7	0.02	0.01	1.24	0.217
	**Age**	21	- 0.03	0.008	-3.72	**0.0002**
	***%* Female**	21	0.01	0.005	2.85	**0.004**
	**Publication's year**	23	- 0.08	0.03	-2.55	**0.011**
	Impact factor	22	0.05	0.06	0.91	0.363
	Quality criteria	23	- 0.0009	0.03	-0.03	0.974
Cognitive bias	Number of training trials per session	20	- 0.0003	0.0004	-0.81	0.420
	***%* Contingency**	20	0.01	0.005	2.92	**0.003**
	CB at baseline	19	0.59	0.50	1.19	0.235
	LSAS at baseline	13	- 0.003	0.005	-0.62	0.537
	BDI at baseline	9	0.006	0.02	0.28	0.782
	STAI-Trait at baseline	7	0.002	0.02	-0.09	0.930
	**Age**	18	- 0.01	0.006	**-**2.15	**0.031**
	*%* Female	18	0.001	0.005	0.19	0.852
	Publication's year	20	- 0.05	0.03	-1.85	0.064
	Impact factor	20	0.03	0.05	0.51	0.610
	Quality criteria	20	- 0.01	0.02	-0.54	0.586
Reactivity in stressful situations	Number of training trials per session	10	0.0006	0.0005	1.22	0.222
	*%* Contingency	10	- 0.008	0.01	-0.74	0.460
	**CB at baseline**	**6**	**0.50**	**0.12**	**4.18**	**0.000**
	LSAS at baseline	7	- 0.0002	0.006	-0.03	0.974
	BDI at baseline	5	0.004	0.05	0.08	0.937
	STAI-Trait at baseline	5	0.02	0.03	0.84	0.399
	**Age**	10	- 0.03	0.02	**-**2.22	**0.027**
	*%* Female	10	0.006	0.009	0.68	0.500
	**Publication's year**	**10**	**- 0.10**	**0.04**	**-2.70**	**0.007**
	Impact factor	10	0.04	0.13	0.31	0.753
	Quality criteria	10	- 0.10	0.07	-1.33	0.181

*Note*: The impact factor of Khalili-Torghabeh (2014) could not be retrieved.

#### Cognitive bias

**Main effect sizes**. The effect of CBM at post-test on CB was small to medium (*g* = 0.44, 95% CI: 0.23 to 0.64, *N* = 24, *Z* = 4.15, *P* < 0.001). However, there was high and significant heterogeneity among the included studies [*Q*(23) = 83.84, *P* = 0.000, *I*^*2*^ = 72.57%]. Four studies [21, 26, 62, 65] were identified as outliers (ESs ≥ 2.5 *SD* from the mean). However, ESs remained significant after the outliers’ removal (*g* = 0.32, 95% CI: 0.21 to 0.43, *N* = 20, *Z* = 5.94, *P* < 0.001) and the heterogeneity became nonsignificant [*Q*(19) = 18.34, *P* = 0.500, *I*^*2*^ = 0.00%]. These outlier studies were excluded from further analyses. We also performed a sensitivity analysis by removing one study at a time; the results did not change with the removal of any one study.

**Subgroup and meta-regression analysis**. Regarding the categorical moderators, the subgroup analysis showed that two moderators had significant effects on CB at post-test (see Table 4). First, regarding the type of bias, ESs for CBM-I were significantly higher than they were for ABM for CB. Second, feedback about training performance also moderated post-test ES; the studies wherein participants received feedback were likely to obtain higher ESs than were those that did not.

**Table 4:** Subgroup analysis for CB at post-test.

Moderator	*N*	*g*	95% CI	*Z*	*P*	*Q*_*w*_	*P*	*Q*_*b*_	*P*
Clinical status									
Diagnosed	8	0.24	0.04 ~ 0.43	2.41	0.016	5.79	0.564	0.89	0.345
Subclinical	11	0.35	0.20 ~ 0.50	4.58	0.000	10.96	0.361		
**Bias**									
**Attention**	**14**	**0.23**	**0.11 ~ 0.36**	**3.61**	**0.000**	**9.88**	**0.703**	**6.65**	**0.010**
**Interpretation**	**6**	**0.54**	**0.34 ~ 0.73**	**5.38**	**0.000**	**1.81**	**0.874**		
Stressor									
Y	8	0.29	0.09 ~ 0.48	2.88	0.004	6.00	0.540	0.19	0.662
N	12	0.34	0.20 ~ 0.48	4.89	0.000	12.20	0.349		
N of training session									
One	6	0.44	0.22 ~ 0.65	4.00	0.000	3.07	0.689	1.46	0.227
More than one	14	0.28	0.17 ~ 0.41	4.36	0.000	13.75	0.392		
Training setting									
Lab	14	0.39	0.25 ~ 0.53	5.35	0.000	12.07	0.522	0.87	0.350
Internet	5	0.26	0.04 ~ 0.49	2.33	0.020	4.12	0.390		
**Feedback**									
**Y**	**7**	**0.49**	**0.31 ~ 0.67**	**5.34**	**0.000**	**3.14**	**0.791**	**5.32**	**0.021**
**N**	**13**	**0.23**	**0.10 ~ 0.36**	**3.47**	**0.001**	**9.88**	**0.626**		

Regarding the continuous moderators, the meta-regression results indicated that two moderating variables were significant for ESs at post-test: participants’ age and the training contingency (see Table 3). More specifically, the younger the participants and the higher the contingency between the cues and probe were, the larger were the ESs.

#### Stressor challenge

**Main effect sizes**. CBM had a small but significant effect on stressor challenge at post-test (*g* = 0.25, 95% CI: 0.02 to 0.49, *N* = 10, *Z* = 2.14, *P* = 0.032); there was no evidence of heterogeneity [*Q*(9) = 15.41, *P* = 0.080, *I*^*2*^ = 41.60%] and no outlier studies. The sensitivity analysis revealed that no specific study drove the results.

**Subgroup and meta-regression analysis**. No categorical moderators were significant (see Table 5). The potential moderating effects of the three categorical variables could not be explored: for type of bias and feedback, it was because only two studies examined the effects of CBM-I and feedback [65, 67], while for training setting, it was because only one study was delivered by the Internet [68]. The degree of heterogeneity within the diagnosed samples was significant, which suggests that there were also several other potential sources of heterogeneity.

**Table 5:** Subgroup analysis for reactivity in stressful situations at post-test.

Moderator	*N*	*g*	95% CI	*Z*	*P*	*Q*_*w*_	*P*	*Q*_*b*_	*P*
Clinical status									
Diagnosed	4	0.18	-0.30 ~ 0.66	0.75	0.455	7.89	0.048	0.28	0.600
Subclinical	6	0.32	0.09 ~ 0.56	2.66	0.008	5.62	0.345		
N of training session									
One	5	0.38	0.04 ~ 0.71	2.19	0.029	7.53	0.110	1.52	0.217
More than one	5	0.10	-0.19 ~ 0.38	0.67	0.504	4.92	0.295		

Meta-regressions revealed that CB at baseline, age of participants, and the year of journal publication moderated post-test ESs for stressor challenge (see Table 3). More specifically, smaller ESs for CB at baseline, and older participants, and more recent publications were associated with smaller ESs.

#### Secondary symptoms

**Main effect sizes**. CBM had a non-significant effect on secondary symptoms at post-test (*g* = 0.03, 95% CI: -0.10 to 0.17, *N* = 19, *Z* = 0.47, *P* = 0.640). The removal of one outlier [69] did not alter the result (*g* = -0.02, 95% CI: -0.12 to 0.09, *N* = 18, *Z* = -0.28, *P* = 0.779), and the heterogeneity was non-significant [*Q*(17) = 14.51, *P* = 0.779, *I*^*2*^ = 0.00%]. The sensitivity analysis revealed that the results were not altered by the removal of any one study.

**Subgroup and meta-regression analysis**. We did not conduct moderator analyses because the general effect was not significant.

### Publication bias

**Fig 3:**
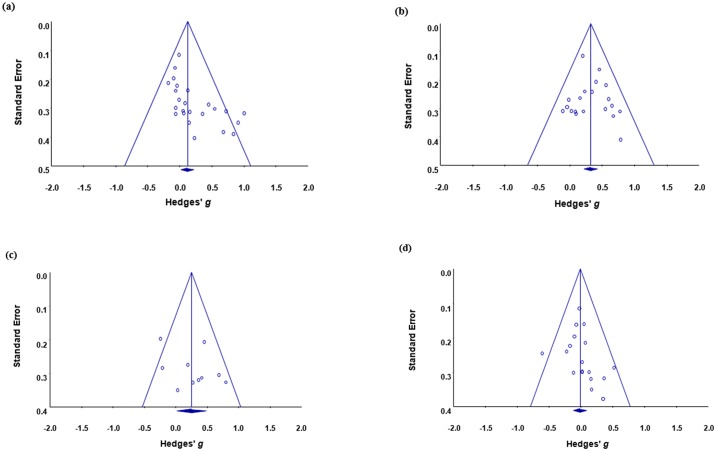
Funnel plots to assess publication bias. (a) SAD primary symptoms; (b) cognitive bias; (c) reactivity in stressful situations; and (d) secondary symptoms.

Publication bias differed for SAD primary symptoms, CB, reactivity in stressful situations, and secondary symptoms at post-test. For SAD primary symptoms, there was no evidence to support publication bias either through examination of the funnel plot shown in Fig 3a or based on the Duval and Tweedie’s trim-and-fill method. Nevertheless, visual inspection of the funnel plots (i.e., Fig 3b, 3c and 3d) showed that there was some evidence of publication bias for the other three dependent measures. For CB, Duval and Tweedie’s trim-and-fill method revealed that one study was missing on the left-hand side. A new mean ES (*g* = 0.31, 95% CI: 0.21 to 0.42, *Q* = 19.73) was imputed by estimating a random-effects model. For reactivity in stressful situations, the trim-and-fill method identified two studies that needed to be trimmed, which decreased the ES to non-significant (*g* = 0.14, 95% CI: -0.09 to 0.38, *Q* = 23.64). For secondary symptoms, the trim-and-fill method indicated that five studies on the left needed to be omitted, resulting in an ES of *g* = -0.07 (95% CI: -0.17 to 0.04, *Q* = 23.56) after adjustment for missing studies.

## Discussion

Given the increase in the body of literature on CBM interventions for SAD and the inconsistent findings in past studies, we thought a quantitative review of studies would be needed. First, we assessed the clinical utility of the CBM procedures for the different types of outcome measure described above. Next, we investigated the possible moderators of treatment response that might have an effect on efficiency of CBM training. Finally, we evaluated the quality of the CBM studies using the “gold standard” tool drawn up by the Cochrane Collaboration to evaluate potential sources of bias. We also evaluated publication bias in two ways: a funnel plot and Duval and Tweedie’s trim-and-fill method.

### Does benign CBM facilitate a reduction in negative cognitive bias and alleviate SAD symptoms?

Our results partly supported and extended the findings from the previous meta-analysis, which examined only the effects of ABM [33]. We found that CBM had a small-to-medium effect on cognitive bias, while it exerted a positive but small effect on SAD primary symptoms and reactivity in stressful situations at post-intervention. Furthermore, in the meta-analysis including all samples, the effects of CBM intervention showed some heterogeneity for all outcome categories except for reactivity in stressful situations. After removal of outliers, ESs significantly decreased across all outcome categories, but still significant. These results contradict a previous meta-analysis by Cristea et al (2015), who concluded that the ESs for reduction of SAD symptoms became non-significant when outliers were excluded and after adjustment for publication bias [34]. The inconsistent findings were likely partly because the two meta-analyses pooled outcomes from different groups: the meta-analysis by Cristea et al included 9 RCTs on social anxiety, of which 6 focused on a clinical sample (after excluding outliers). In contrast, our meta-analysis included 24 RCTs on SAD primary symptoms, of which 13 focused on a clinical sample after outliers were excluded. Heeren (2015) further suggested that the clinical efficacy and varying moderators (e.g., participants’ age, number of sessions, type of threatening stimuli used during training) might be the main cause of such inconsistencies [13]. The current findings suggested that the clinical therapeutic efficacy for a reduction in the symptoms of SAD produced by CBM was mild. There are several possible explanations for these results. First, in order to enrich cognitive theories of SAD and enhance the availability of CBM programs, researchers frequently test new variations of CBM by varying the tasks, experimental materials, instructions, number of sessions, etc. Such attempts might generate nonsignificant ESs or even negative ones, and consequently, these data might contribute to the attenuation of the total ES when we pool the results from the relevant studies to evaluate the efficacy of CBM treatment for SAD. Second, the reinforcement sensitivity theory (RST) proposes a unified model of SAD that integrates a wide range of factors and suggests that CB is just one of the most important factors; it is not, in other words, a uniquely crucial factor. Indeed, many factors in the model (i.e., genetic, biological, temperamental, and environmental factors) are simultaneously associated with the development and maintenance of SAD [75,76]. This standpoint is at odds with cognitive theories of SAD, which suggest that cognitive bias contributes significantly to the onset, development, and maintenance of SAD [77–79]. Third, Clark suggested that social anxiety was characterized by a diverse range of biases in information processing [80]. However, most CBM interventions only target one type of negative CB (i.e. attention bias) and the effects were not optimal, meaning that the other types of CB would not be effectively modified. Furthermore, in some studies, even the target CB could not be successfully modified, despite the CBM having an effect on SAD symptoms by modifying an initial negative CB [81]. As a result, the ESfor the reduction of SAD primary symptoms would be increased by filtering those studies that failed to alter the bias. A recent meta-analysis of interventions for adults with SAD uncovered the interesting finding that a number of psychological treatment methods, including psychodynamic psychotherapy, interpersonal psychotherapy, cognitive behavioral therapy (CBT), mindfulness, and supportive therapy, were all effective to varying degrees in reducing symptoms [82]. This might partly confirm the famous “dodo bird verdict,” which proposes that all psychotherapies have equal effects [83]. Psychological treatment specific to SAD is no exception. It would make sense, then, that each type of psychological intervention for SAD, including CBM, merely addresses certain aspects of a sufferers’ problems. Relatedly, some studies have found that combining CBM with CBT could enhance the training effects on social anxiety [68,84,85]. This hints that CBM might be a beneficial complement to traditional methods of psychotherapy.

### How do we enhance the effectiveness of benign CBM?

As previous findings have indicated [34,35], we found that ABM had smaller ESs for SAD symptoms and CB at post-test than did CBM-I; in fact, the effect on SAD symptoms was nonsignificant. This difference might be explained from an information processing perspective, in which AB relates more to automatic processing systems whereas interpretative bias relates to more conscious, strategic processes [39,86]. Furthermore, the content of CBM training materials mostly feature various social situations (i.e., public speaking and meeting strangers), which are more specific to social anxiety. It might be that CBM-I interventions have a more powerful influence on deeper cognitive mechanisms (i.e., dysfunctional cognitive schemas) that possibly underlie these biases, rather than merely temporarily “switching off” CBs. Additionally, a qualitative study about socially anxious individuals’ attitudes toward CBM found that participants reported greater understanding and engagement with the CBM-I program than with the ABM program [87]. Intriguingly, recent research has shown that, for social anxiety, benign ABM did not excel in improving symptoms compared to placebo control procedures [27,32,42,45,56,57,66,68]. A recent study further suggested that adding a negative intervention as a control group might be more beneficial for ABM specific to social anxiety [34]. We agree with them and argue that it was not difficult to find that almost all of the above ABM interventions that did not show an advantage were delivered remotely.

The present meta-analysis showed that ABM programs conducted in a laboratory yielded significantly larger ESs for SAD primary outcomes compared with ABM programs conducted via the Internet. The two previous meta-analyses had similar results [33,37], consistently reporting greater SAD symptom reductions in the laboratory condition than in the remote condition. Previous researchers [57,88] have pointed out that participation in a relatively unconstrained home-based setting might be subject to external distractions, thus reducing the benefits of Internet-delivered procedures. Emmelkamp even suggested that it might be necessary to keep a watchful eye on ABM delivered via the Internet [89]. Later on, two scholars commented on this inference and believed that more research in this field is necessary [90,91]. We agree with the latter and makeseveral recommendations for future CBM studies on SAD that will further enhance our understanding. First, it would be necessary to formulate more rigorous guidance (just as for internet-delivered CBT, see [92]) for participants receiving CBM interventions delivered by the Internet, which would help guarantee consistency and quietness in the treatment environment without distractors. Second, it would help to make the CBM tasks more understandable and engaging in order to offset the adverse effects produced by distractors. Finally, it would be important to evaluate the reliability of CBM delivered remotely in naturalistic settings in future research.

The meta-regression analysis showed that a higher percentage of training contingency between the location of benign cues and the probe in CBM was associated with larger CB reduction. There should be a dose–response gradient between these two variables, supposing that the percentage of contingency is defined as the active dose that might direct attention disengagement from the threatening stimuli delivered to participants. These findings are not quite the same as those reported in previous meta-analyses [33,93,94], that regarded the number of CBM sessions completed as the dose. Although conceptual discrepancies concerning the dose might exist, the current findings suggest that future CBM studies should include a higher training contingency, as this is likely to lead to greater effects on reducing CB and SAD symptoms.

Although studies in which participants were or were not provided feedback during training both have significant effects on CB reduction, the results herein suggest that the presence of feedback to participants yielded larger ESs than the absence did. Consistent with Menne-Lothmann et al.’s meta-analysis [36], feedback on response accuracy might encourage participants to engage in and concentrate on the information presented. In this way, participants might receive greater assistance in decreasing the negative CB compared with the sham condition [25,95].

### Do relatively susceptible populations benefit more from CBM?

The meta-regression results suggest that benign CBM was particularly effective for women. In line with a previous meta-analysis [36], studies that included a higher percentage of female participants tended to show higher ESs for the reduction in SAD symptoms. In line with the “gender differences in processing content” hypothesis [96], some studies have showed that gender moderates the relationship between CBs and anxiety [97–99], while there is some evidence that women showed greater processing of stimuli than did men at the early attention stage in anxious arousal states [100]. A previous study found gender differences in social anxiety, specifically, more women than men reported greater fear regarding going to a party or speaking at a meeting, whereas more men than women reported greater fear regarding urinating in a public restroom [101]. Current CBM procedures mainly adopt emotional faces or words or social scenarios as experimental materials, which might be more beneficial to female participants. Future CBM interventions could take gender differences into consideration to ensure more obvious therapeutic effects.

The present meta-analysis also examined the relationship between participants’ age and the efficacy of CBM across most outcome categories, and we consistently found that younger participants benefited more from CBM interventions than did older ones (see [33,34]). One researcher concluded that when healthy and educated adults are in their twenties and thirties, they begin exhibiting declines in certain age-related cognitive abilities [102]. It is possible that cognitive reappraisal and executive function, which are associated with the prefrontal cortex, are highly vulnerable to age-related declines [103,104]. Thus, cognitive reappraisal, an important contributor to emotion regulation (which itself requires intact cognitive control ability), might be more successfully used by younger participants. Conversely, age-related deficits in executive processes involving attention, switching, inhibition, and working memory might lead to older participants’ poorer performance compared to that of younger participants on a series of cognitive tasks [105].

Interestingly, we also found that greater CB at baseline within studies seemingly yielded stronger ESs for reactivity in stressful situations at post-test. Similarly, Mogoașe found that AB at baseline correlated significantly with a change in AB post intervention [37]. However, we cannot be sure that there was a “true” baseline CB in the groups receiving CBM intervention. Therefore, these results must be cautiously interpreted.

### Did the risk of publication bias exist in the current meta-analysis?

The quality of the included studies, as assessed by the Cochrane Collaboration Risk of Bias Tool (CCRBT), was substandard. More specifically, half of the studies satisfied fewer than three quality standards. Moreover, a varied proportion of the included studies did not provide information necessary for judging the five risks of bias (ranging from 5.6 to 41.7%).

However, positively biased selection was small or even non-existent for some of the outcomes, owing to negative or null findings included in the meta-analysis. The meta-regression results indicated that there were significant negative linear relationships of publication year with SAD symptom reduction and reactivity in stressful situations. Consistent with a previous meta-analysis [34], more recent experiments had smaller ESs than did earlier ones. As in other types of intervention research, CBM is an innovative approach, which means that it is likely subject to publication bias—that is, “positive” and statistically significant studies are likely to be published more rapidly than are studies with “negative” and statistically non-significant results [106–108]. Thus, we could not agree more with the suggestion that, to avoid publication bias, psychotherapy researchers should prospectively register all trials before their results are known.

### Limitations and future directions

There are several limitations to the present meta-analysis. First, the apples-and-oranges problem persists in this study, which is a common critique of meta-analyses [109]. More specifically, the results from different studies that measured different variables and described different populations were combined statistically in this meta-analysis. Admittedly, there was some evidence of heterogeneity for SAD symptoms and CB at posttest. However, the removal of outliers reduced the heterogeneity to non-significance for the main outcome categories. Nonetheless, when we conducted the subgroup analyses to examine differences between varying study features, *I*^*2*^ remained large for a few outcomes, indicating that heterogeneity remained. This is possibly because of the different types of measures used in CBM studies, which we were only able to classify roughly. Second, there was some evidence-based publication bias in the post-test data set. Often, studies with nonsignificant results are less likely to be published than are studies with significant results. To this end, we contacted authors and requested unpublished data if published articles lacked the essential data to calculate an ES. The results did not change after taking these steps. Third, on the whole, the quality of the included studies was suboptimal, with only half of the included studies meeting three of the five quality criteria and two studies meeting none. Moreover, some studies did not report sufficient information for assessing whether the quality criteria were satisfied, which further increases bias. Finally, it was impossible to compute categorical moderation analyses, as some subgroups only included a small number of studies (i.e., different types of training setting when investigating stressor challenges at post-test).

In view of our finding that CBM had small effect sizes on SAD symptoms, future CBM research could be developed in several ways, each of which also has potentially important theoretical or clinical implications. First, as stated earlier, future research could explore the crucial characteristics that might enhance the effectiveness of CBM, especially in real-world settings, and clarify the functional relationship between CBM and different facets of SAD symptomatology. Recently, a review on the neural effects of CBM for anxiety, addiction, and depression was published in *NeuroImage*, which included a total of 13 published studies that used (functional) magnetic resonance imaging (MRI) or electroencephalography as an outcome measure; 5 of these studies were on social anxiety [110]. Future studies could thus adopt some techniques commonly used in cognitive neuroscience (i.e., fMRI) to explore the possible neurobiological basis of CB in SAD. Additionally, future research could refine the therapeutic applications of CBM procedures during clinical interventions for SAD in order to optimize their capacity to change target CBs and reduced SAD symptoms. Second, we found that CBM-I was more successful for modifying CB and reducing SAD symptom. Preliminary data indicated that CBM-I could not only guide participants’ interpretation in positive directions, but also has the ability to disengage attention from threat stimuli [55]. This notion is compatible with the combined CB hypothesis [111], which suggested that a change in interpretative bias might influence other aspects of cognitive processing (i.e., attention bias); that is, different forms of CB might interact with each other to maintain social anxiety. Future CBM research specific to SAD must verify the complicated cognitive mechanism underlying SAD and show how variants of CBM program targeting one CB influence other types of bias and then reduces social anxiety. Third, we think that the cultural context of participants exhibiting social anxiety should be taken into account in future CBM interventions. Specifically, Asian individuals living in collectivistic cultures, which emphasize on harmony within the group, tend to have a more interdependent self-construal. In contrast, individuals with a European heritage living in individualistic cultures that value individual achievements and success, tend to possess a more independent self-construal [112,113]. Hence, future studies should examine cultural differences in CBM interventions for social anxiety.

## Supporting information

S1 Table PRISMA checklist. (DOC)

S2 Table Coding of the four categories for the dependent measures. (DOC)

S1 Text Example of the search strategy. (PDF)
