# Supplementary material for: Effects of cognitive bias modification on social anxiety: A meta-analysis
Source: PLoS One. 2017 Apr 6;12(4):e0175107. doi: 10.1371/journal.pone.0175107 (PMC5383070; doi:10.1371/journal.pone.0175107)
Supplement: S2 Table — (DOC) [file pone.0175107.s002.doc]

**S2 Table. Coding of the four categories for the dependent measures.**

**Table S2** Coding of the four categories for the dependent measures

| Outcome categories | Measures |
| --- | --- |
| SAD core symptoms | BARS;BSPS; FNE; LSAS; PRCS; SADS; SASC; SIAS; SIPS; SPAI; SPIN; SPS; SPSQ |
| Cognitive bias | Dot-probe task; Spatial cueing task; WSAP; Ambiguous social scenarios |
| Reactivity in stressful situations | BASA; IST; PSP; STAI-S; SUDS; SPRS; UCT; VAS |
| Secondary symptoms | BAI; BDI-II; CDI; CES-D; CGI; DASS; HAM-D; IPSM; MADRS; PANAS; PSWQ; QoLI; RCADS; STAI-T |

Notes. BARS = Behavioral Avoidance Rating Scale; BSPS = Brief Social Phobia Scale; FNE = Fear of Negative Evaluation; LSAS = Liebowitz Social Anxiety Scale; PRCS = Personal Report of Conﬁdence as a Speaker; SADS = Social Avoidance and Distress Scale; SASC = Social anxiety scale for children; SIAS = Social Interaction Anxiety Scale; SIPS = Social Interaction Phobia Scale; SPAI = Social Phobia and Anxiety Inventory; SPIN = Social Phobia Inventory; SPS = Social Phobia Scale;SPSQ = Social Phobia Screening Questionnaire; AAT = Approach-Avoidance Treatment; WSAP = word–sentence association paradigm; BASA = Behavioral Assessment of Speech Anxiety; IST = Impromptu Speech Task; PSP = Perception of Speech Performance; STAI-S/STAI-T = State-Trait Anxiety Inventory-State/State-Trait Anxiety Inventory-Trait; SUDS = Subjective Units of Discomfort Scale; SPRS = Social Performance Rating Scale; UCT = Unstructured Conversation Task; VAS = Visual Subclinical scales; BAI = Beck Anxiety Inventory; BDI-II = Beck Depression Inventory-II; CDI = Children’s depression inventory; CES-D = Centre for Epidemiological Studies Depression Scale; CGI = Clinical Global Impression of Improvement; DASS = Depression, Anxiety, and Stress Scale; HAM–D = Hamilton Rating Scale for Depression; IPSM = Interpersonal Sensitivity Measure; MADRS = Montgomery–Asberg Depression Rating Scale; PANAS = Positive and Negative Affect Schedule; PSQW= Penn State Worry Questionnaire; QoLI = Quality of Life Inventory; RCADS = The Revised Child Anxiety and Depression Scale
