# Supplementary material for: Effects of cognitive bias modification on social anxiety: A meta-analysis
Source: PLoS One. 2017 Apr 6;12(4):e0175107. doi: 10.1371/journal.pone.0175107 (PMC5383070; doi:10.1371/journal.pone.0175107)
Supplement: S1 Text — (PDF) [file pone.0175107.s003.pdf]

S1 Text. Example of the search strategy.

1．social phobi*.mp. or exp social phobia
2．social anti*.mp
3．1 or 2
4．cognitive bias modification.mp
5．attention* bias modification.mp
6．interpret* bias modification.mp
7．attention* training.mp
8．bias experimental manipulation.mp
9．bias training.mp
10．4 or 5 or 6 or 7 or 8 or 9
11．3 AND 10
